# Analysis of the Volatile Profile of Core Chinese Mango Germplasm by Headspace Solid-Phase Microextraction Coupled with Gas Chromatography-Mass Spectrometry

**DOI:** 10.3390/molecules23061480

**Published:** 2018-06-19

**Authors:** Xiao-Wei Ma, Mu-Qing Su, Hong-Xia Wu, Yi-Gang Zhou, Song-Biao Wang

**Affiliations:** Ministry of Agriculture Key Laboratory of Tropical Fruit Biology, South Subtropical Crops Research Institute, Chinese Academy of Tropical Agricultural Sciences, Zhanjiang 524091, China; maxiaowei428@126.com (X.-W.M.); pillar1984@163.com (M.-Q.S.); whx1106@163.com (H.-X.W.); zhouyigang@21cn.com (Y.-G.Z.)

**Keywords:** mango germplasm, volatile compound, HS-SPME-GC-MS, multivariate analysis

## Abstract

Despite abundant published research on the volatile characterization of mango germplasm, the aroma differentiation of Chinese cultivars remains unclear. Using headspace solid phase microextraction (HS-SPME) coupled with gas chromatography–mass spectrometry (GC-MS), the composition and relative content of volatiles in 37 cultivars representing the diversity of Chinese mango germplasm were investigated. Results indicated that there are distinct differences in the components and content of volatile compounds among and within cultivars. In total, 114 volatile compounds, including 23 monoterpenes, 16 sesquiterpenes, 29 non-terpene hydrocarbons, 25 esters, 11 aldehydes, five alcohols and five ketones, were identified. The total volatile content among cultivars ranged from 211 to 26,022 μg/kg fresh weight (FW), with 123-fold variation. Terpene compounds were the basic background volatiles, and 34 cultivars exhibited abundant monoterpenes. On the basis of hierarchical cluster analysis (HCA) and principal component analysis (PCA), terpinolene and α-pinene were important components constituting the aroma of Chinese mango cultivars. Most obviously, a number of mango cultivars with high content of various aroma components were observed, and they can serve as potential germplasms for both breeding and direct use.

## 1. Introduction

Sweetness, sourness and aroma constitute the main components of fruit flavour, with aroma being the most important contributing factor [1]. With the increasing requirement for fruit table quality and quality of processed products, fruit aroma has gained increasing research attention in recent years. Aroma components in fruits mainly consist of aldehydes, alcohols, esters, lactones, ketones, quinones and terpenes [2,3]. Each of these volatile compounds has a distinct odour, and their combinations, concentrations and ratios confer unique aroma characteristics to different fruits through cumulative, synergistic and masking effects [4]. The concentrations and composition of volatile compounds in fruits, although influenced by climatic and cultivation conditions [5,6,7], are mainly determined by the genetic background of the plants [8,9]. Therefore, the evaluation of volatile aroma compounds in fruits at the germplasm level is essential.

Mature mango possesses a rich flavour, which is a key characteristic which attracts consumers. Studies have indicated that mango aroma is the result of a mixture of terpenoids, alcohols, aldehydes, carbonyl compounds, esters, nitrogen-containing compounds, and other volatiles, with the composition and content of these aroma compounds in different cultivars being significantly distinct. At present, studies on aroma compounds in mango fruit at the germplasm level have focused on cultivars from India, Australia, the United States, Brazil and Cuba [10,11,12,13]. However, there are limited reports on the volatile profile of mango fruits at the germplasm level from China, which is an important producer of mango producing 129 million tonnes in 2013 according to the Food and Agriculture Organization.

In China, over the past 30 years, breeding objectives for mango have been primarily associated with yield, resistance, and appearance, with less emphasis on the improvement of flavour-associated traits such as the fruit aroma. As a result, superior flavour traits originally present in germplasm resources have been gradually lost during breeding, thus leading to largely similar fruit aroma amongst current commercial cultivars. At present, China’s mango germplasm collection comprises 200 cultivars, and aroma sensory evaluations have revealed a marked variation in fruit flavour among these different cultivars which this affords the possibility of selecting potential parents for hybrid breeding. Phenotypic diversity assessment of fruit quality traits, for instance aroma, was the first step for effective germplasm conservation and utilisation. Although some literatures are available in the field of mango aroma, they are generally limited to particular cultivars [14].

In this study, 37 representative Chinese mango cultivars encompassing different maturation periods (early, moderate and late maturation), colour types (green, yellow and red) and genetic parents were selected as study materials [15,16]. The characteristics of volatile compounds in the fruits of different mango cultivars were studied using headspace solid-phase microextraction (HS-SPME) in conjunction with gas chromatography–mass spectrometry (GC-MS) with the aim of understanding characteristics of “good” mango fruit for ultimate flavour improvement.

## 2. Results and Discussion

### 2.1. Identity and Concentration of-Volatile Compounds in Mango Cultivars

A total of 114 volatile compounds in the pulp were identified and relatively quantified, some of which were found only in a few of the cultivars in this study (see Table 1, Table 2, Table 3, Table 4, Table 5, Table 6 and Table 7). These compounds included 23 monoterpenes, 16 sesquiterpenes, 29 non-terpene hydrocarbons, 25 esters, 11 aldehydes, five alcohols and five ketones. 

**Figure 1:**
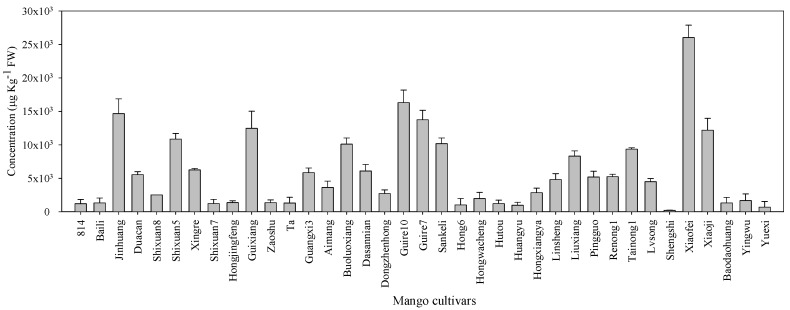
Concentrations (μg/kg fresh weight (FW) equivalent of nonyl acetate) of total volatile in 37 mango cultivars.

Monoterpenes, non-terpene hydrocarbons and esters were detected in all 37 cultivars, whereas most cultivars had no aldehydes, alcohols and/or ketones. The total volatile content showed great variation in different mango cultivars, and it ranged from 211.01 μg/kg fresh weight (FW) in Shengshi to 26,021.91 μg/kg FW in Xiaofei (Figure 1). The total aroma contents in Xiaofei, Guire 10, Jinhuang, Guire 7, and Guixiang cultivars were significantly higher than in the other cultivars. The fruit aroma is closely related to the content and number of volatile compounds. Significant differences of the number of volatiles were also found amongst the 37 cultivars. Xiaofei fruits contained the greatest number of volatiles (49), followed by Boluoxiang (48) and Tainong 1 (43), whereas Zaoshu had the least, with only 17 volatile compounds being detected. In previous sensory perception tests, Xiaofei fruit has been evaluated to exhibit obvious characteristics of aromatic flavour, which is associated with rich compounds and relatively high content of volatiles.

### 2.2. Relative Abundance of Different Classes of Volatile Compounds

**Figure 2:**
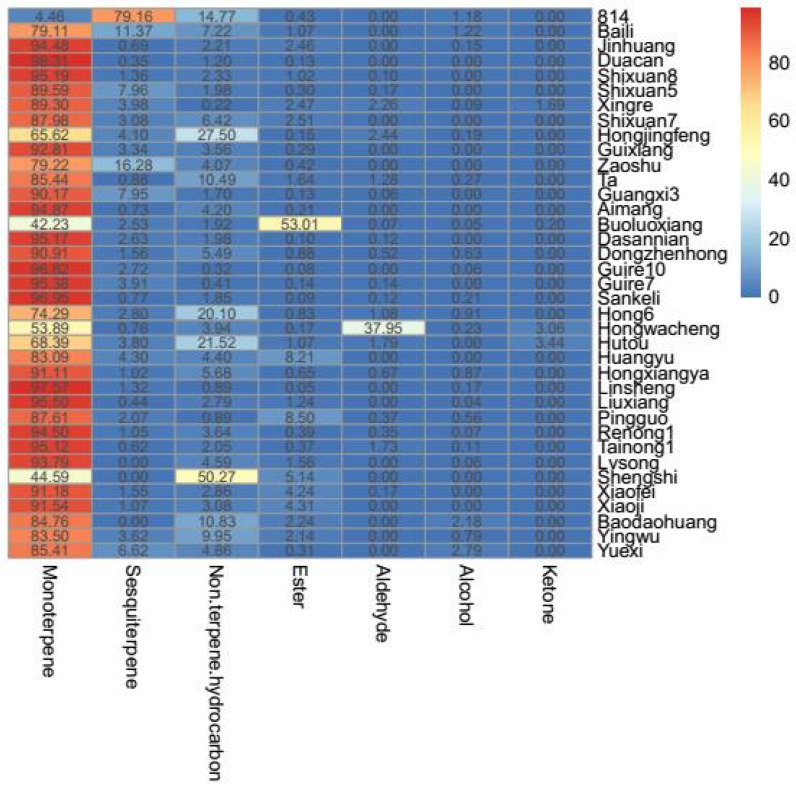
Relative abundance (%) and heatmap of different classes of volatile compounds in 37 mango cultivars.

Variation in the relative abundance of terpenes, non-terpene hydrocarbons, esters, aldehydes, alcohols and ketones amongst different mango cultivars was significant (Figure 2). Amongst the 37 mango cultivars examined, non-terpene hydrocarbons were the major volatiles in Shengshi, esters were the dominant volatiles in Boluoxiang fruit, while monoterpenes and sesquiterpenes were the dominant volatiles in other cultivars. Terpenoids are synthesised via two alternative pathways: the cytosolic mevalonate pathway and the plastidic methylerythritol-4-phosphate pathway [17]. Previous studies have shown that terpenoids, particularly monoterpenes, form the predominant volatile compounds in mango fruits [18,19]. Based on the content of terpenoids, Andrade [13] divided Brazilian mango varieties into three groups: group 1 with abundance of terpinolenes, group 2 with abundance of 3-carenein, and group 2 with abundance of myrcene. In the present study, Xiaofei fruit had the highest monoterpene content, with 23,726.61 μg/kg, whereas cultivar 814 had the lowest content with only 54.17 μg/kg FW. Monoterpenes accounted for 65.62–98.31% of the total concentrations of volatiles in all cultivars except for 814, Shengshi and Boluoxiang. In addition, α-pinene and terpinolene were identified in all cultivars and were considered to be important volatile components. Pingguo contained the highest level of α-pinene (1661.56 μg/kg FW; 31.62% of total volatiles), whereas Guire 10 had the highest terpinolene content (12,725.64 μg/kg FW; 78.04% of total volatiles) (see Table 2).

No sesquiterpenes were detected in Lvsong, Shengshi and Baodaohuang cultivars. In 34 other cultivars, 814 mango had high total sesquiterpene content (961.96 μg/kg; 79.16% of total volatiles) mainly due to the high content of caryophyllene, α-caryophyllene and α-selinene. However, TA showed the lowest sesquiterpene content (11.66 μg/kg; 0.88% of total volatiles). Of the 26 sesquiterpenes, caryophyllene and α-caryophyllene were detected in 32 and 25 cultivars, respectively (see Table 3).

Significant differences of terpene constituents were also found amongst the cultivars. For example, Guixiang had a very high content of limonene (10,565.52 μg/kg), but 3-carene and β-piene were not detected. In Boluoxiang, 3-carene and 3,7,7-trimethyl-bicyclo[4.1.0]hept-3-ene were the dominant volatiles, but limonene was not detected. Liu et al. [14] detected selinene, eremophilene and aromadendrene only in Jinhuang mango, cinene only in Irwin and caryophyllene in Keitt. Fruit odours can be classified as fruity, green, spicy, woody and aldehydic based on sensory responses to aroma components with different chemical structures [20,21]. Odour components with different chemical structures elicit different sensory perceptions. For example, ocimene has a grassy odour, 3-carene has a sweet odour and cinene has a soft lemon odour [18]. Odour differences amongst mango cultivars may be associated with differences in terpenoid-type odour components.

Significant differences were found in both the total content and number of non-terpene hydrocarbons amongst different mango cultivars (see Table 4). The sum content of non-terpene hydrocarbons ranged from 13.60 μg/kg (Xingre) to 744.13 μg/kg (Xiaofei). Amongst all cultivars, Hongjinfeng, Guixiang and Hutou were rich in non-terpene hydrocarbons, with 20, 18 and 18, respectively. Non-terpene hydrocarbons were the only predominant volatile compounds in Shenshi and represented 50.27% of the total volatiles. Meanwhile, for the remaining cultivars, the content of non-terpene hydrocarbons was low and accounted for less than 28% of the total volatiles.

Previous studies have shown that aldehydes are present at low concentrations, account for a small percentage of total volatiles in mango fruits and are important to mango flavour and aroma [22,23,24]. In this regard, Pino et al. [18] reported that the green, grassy odour of Cuban mangoes is derived primarily from aldehydes. In our study, aldehydes were present only in 19 cultivars, wherein their concentrations ranged from 2.42 μg/kg FW to 747.40 μg/kg FW (0.06–37.95% of total volatiles) in mango fruits. Amongst all cultivars studied, Hongwacheng with a sweet, fatty and green odour had the highest aldehyde content, particularly furfural (168.90 μg/kg FW) and 5-methyl-2-furancarboxaldehyde (566.11 μg/kg FW) (see Table 5).

Aldehydes are usually converted into alcohols or carboxylic acids, which are further converted into esters [25]. The present study could not determine which alcohols or esters were derived from aldehydes due to experimental limitations. Although certain alcohols are odour molecules, they do not substantially contribute to odour perception due to their higher odour threshold values compared with homologous aldehydes. A variety of esters are formed upon association of different alcohols with acetyl coenzyme A [26,27,28]. Aliphatic esters, which are mainly synthesised in actively growing tissues, are responsible for the odours of nearly all fruits. Lactones have classic fruity odours and are the second most abundant volatile aroma compounds in mangoes [29,30]. In this regard, Wilson et al. [31] asserted that esters in fruits could be detected at very low levels, and the contribution of lactones to mango aroma was second only to that of terpenoids. Even though esters are important odour components in mangoes, there is a large variation in the composition of esters amongst fruits of different origins and cultivars. In this regard, Pino detected 90 aliphatic, 16 aromatic and eight terpene esters from 20 Cuban mango cultivars. Amongst them, ethyl acetate and ethyl butanoate were the dominant ones. Eight lactones were found in 22 Indian and five non-Indian cultivarsbut they represented less than 1% of the total volatiles, and γ-butyrolactone was the major component [4]. In the present study, 24 aliphatic esters were detected in the mango germplasm, and no lactones were found. In different cultivars, the total contents of ester compounds varied widely, ranging from 2.01 μg/kg FW to 5361.09 μg/kg FW. Interestingly, Boluoxiang, which exhibited a characteristic fruity smell, had the highest content of esters (5361.09 μg/kg FW) and accounted for 53.01% of the total volatiles. Butanoic acid ethyl ester and octanoic acid ethyl ester were the major compounds in Boluoxiang (2916.45 and 1699.42 μg/kg FW, respectively). However, other cultivars had relatively low concentrations of ester compounds, i.e., 0.05–8.21% of the total volatiles (see Table 6).

The components and contents of alcohols and ketones in mango fruits are low [23,24]. Consistent with this trend, only five alcohols were found in 22 cultivars in this study. Alcohol concentrations ranged from 2.66 μg/kg FW to 29.22 μg/kg FW and accounted for 0.04–2.79% of the total volatiles. Ketone compounds were only found in Xingre, Boluoxiang, Hongwacheng and Hutou, with concentrations of 105.51, 19.92, 60.27 and 42.00 μg/kg FW, respectively (see Table 7).

### 2.3. Relationships Among Different Classes of Volatile Compounds

Correlation analysis was used to explore the relationships amongst different classes of volatile compounds (see Table 8). Sesquiterpene and non-terpene hydrocarbons were highly correlated with total monoterpene content (r = 0.374 and 0.569, respectively). Aldehydes were highly correlated with ketone content (r = 0.565, *p* < 0.01). Such correlation can facilitate the selection of cultivars with improved aroma quality because selection for one trait leads to the selection of genetically correlated traits. However, no significant relationships were found between aldehydes, esters and alcohols. The relationships amongst different classes of volatile compounds showed the complexity of fruit aroma metabolites.

### 2.4. Multivariate Data Analysis

**Figure 3:**
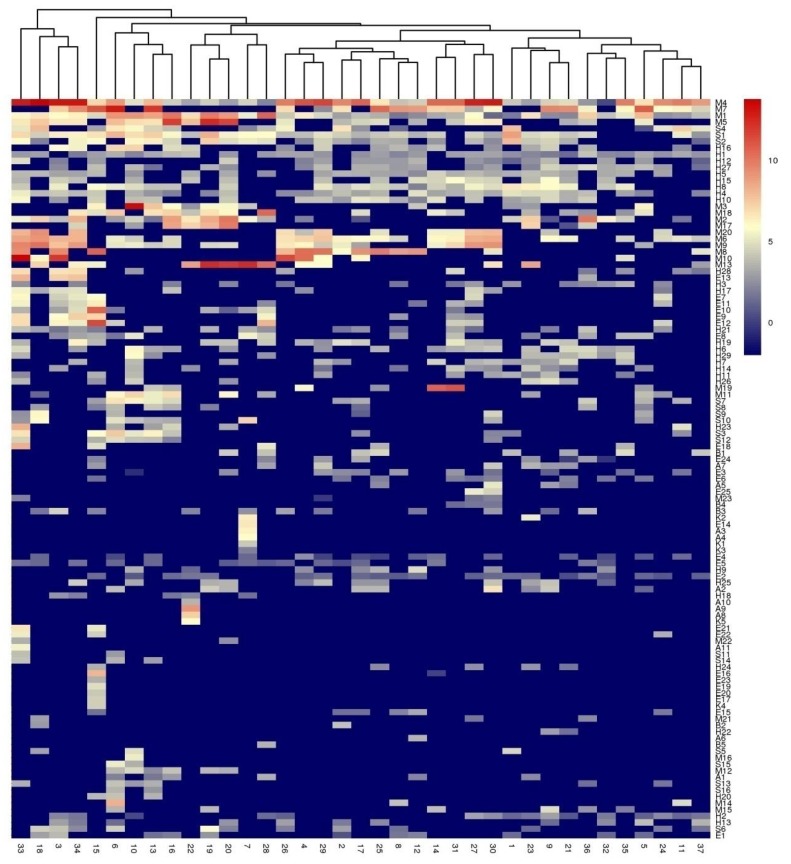
Hierarchical clustering (HCA) and heatmap of volatile compounds levels in 37 mango cultivars. values of all studied volatile compounds per cultivar are shown in the heatmap on a blue (negative) to red (positive) scale. The HCA and dendrogram of cultivars was according to Euclidean distance.

Hierarchical cluster analysis (HCA) was used to analyse the data of 114 volatile compounds obtained from 37 mango cultivars (Figure 3). Thirty-seven mango cultivars could be divided into four groups. The first group included four cultivars: Xiaofei (33), Guire 10 (18), Jinhuang (3) and Xiaoji (34), which were characterized as having high concentrations of terpinolene (M4), δ-terpinolene (M9), α-terpinolene (M6), α-pinene (M1) and β-myrcene (M5). The second group included only one cultivar Boluoxiang (15) with high concentrations of butanoic acid ethyl ester (E12), 3-carene (M7), (1S)-3-carene (M8), octanoic acid ethyl ester (E10), limonene (M3) and α-pinene (M1). This cultivar was characterized by an extremely high esters content. The third group contained nine cultivars with high concentrations of α-pinene (M1), terpinolene (M4) and β-myrcene (M5). The other 23 cultivars were classified into group four. However, determining the dominant volatiles in this group was difficult. Although terpinolene and α-pinene were detectable in these cultivars, they were present only at very low levels except for 13 cultivars with high concentration of terpinolene. Thus, no characteristic volatile was observed for this group.

**Figure 4:**
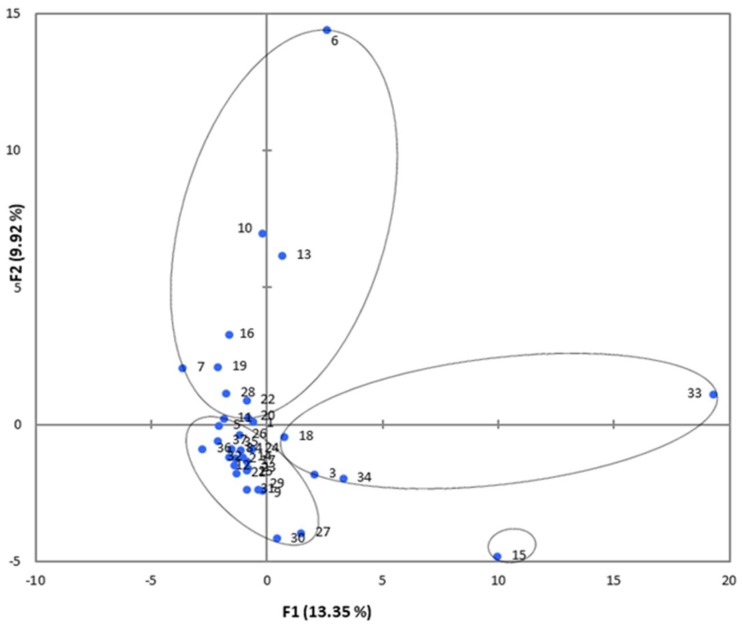
Positions of PC scores of all of the studied mango cultivars according to PC1 and PC2.

To further elucidate genetic clustering identified by HCA, we performed—principal component analysis (PCA) (Figure 4). However, the cumulative contribution of the first 14 components was only 80% (data not shown). This indicates the presence of relatively large variations in the composition and concentration of aromatic compounds in different cultivars, which results in scattered contribution rates of various aroma compounds and an insignificant cumulative contribution rate. Although two principal components (PC1 and PC2) represented only 23% of the variability, the mango germplasms can be divided into four groups based on the score scatter plot shown in Figure 3. In general, the PCA results were in accordance with the results of HCA.

Based on this study, terpinolene and α-pinene are the main volatile compounds responsible for Chinese mango aroma. The contributions of different volatile aroma compounds to fruit aroma are affected by the odour activity value, flavour dilution factors and aroma profile [32,33]. Given that the present study only analysed the content of aroma compounds, further studies are required to determine whether certain compounds act as characteristic aroma compounds in mangoes.

## 3. Materials and Methods

### 3.1. Materials

The 37 cultivars (see Table 9) considered in this study were cultivated in the orchards of the South Subtropical Crops Research Institute in Zhanjiang, China. All cultivars were grown under the same geographical conditions and with the same standard cultural practices. Based on production experience and days after pollination, the fruit of 37 cultivars were harvested at commercial maturity (with the flesh around the seeds starting to turn yellow) from June to July 2017. Three trees with moderate growth vigour were selected for each cultivar. Ten disease-free fruits of similar sizes were randomly picked from different locations on the crown of each tree and considered to be one replication, thus resulting in three replications per cultivar. All the fruits sampled were stored under a controlled atmosphere with day and night temperatures of 24 °C and 18 °C, respectively, for ripening, which was ascertained for each cultivar by conventional indices such as change in skin colour, smell, and softness to touch. Ripe fruits were peeled immediately, four slices were then taken by longitudinal cuts from different orientations of each fruit, and ground to powder in liquid nitrogen and stored at −70 °C for further studies.

### 3.2. Methods

#### 3.2.1. Volatiles Extraction

For HS-SPME, the extraction of aroma volatiles was performed using an SPME fibre coated with polydimethylsiloxane-divinylbenzene (65 μm) (Supelco, Bellefonte, PA, USA). The fibre was preconditioned for 30 min per day at 250 °C according to the manufacturer’s instructions. For each extration, 8 g pulp, 2 g NaCl and 30 μL internal standard (0.29 μg/mL nonyl acetate) were placed in a 20 mL capped SPME vial. The mixture was incubated in a water bath at 40 °C for 10 min with a magnetic stirrer. Next, the fibre was exposed for 40 min to the headspace. After extraction, the fibre was immediately inserted into the heated chromatograph injector port for desorption at 250 °C for 2 min in the splitless mode.

#### 3.2.2. GC-MS Analysis

The volatile compounds were analysed by means of an Agilent 6890N gas chromatograph coupled with an Agilent 5973N mass selective detector (Agilent, Santa Clara, CA, USA) and equipped with a DB-5 MS (Supelco, Bellefonte, PA, USA) capillary column (30 m × 0.25 mm ID × 0.25 μm film thickness). The injector and detector temperatures were maintained at 220 °C and 250 °C. The oven temperature program were as follows: 50 °C for 1 min, increased at 5 °C/min to 140 °C, then increased at 10 °C/min to 250 °C and then kept for 10 min. Mass spectra conditions were as follows: electron impact mode at 70 eV, ion source temperature: 250 °C, mass scanning range: *m*/*z* 35–335 amu/s. The carrier gas was helium with a constant column flow of 1 mL/min [12,13]. The tentative identification of the volatile compounds was done by comparing the mass spectra with the data system library (NIST98) and linear retention index. Using a series of *n*-alkane standards (C8–C29), retention indices of each compound were determined. Semiquantitation was done by the internal standard method, where the relative content of each volatile compound was obtained as nonyl acetate equivalent by the GC peak area.

#### 3.2.3. Data Analysis

Data for each cultivar were averages of three replication. Hierarchical cluster analysis (HCA) and principal component analysis (PCA) were carried out to detect clustering and establish relationships between cultivars and volatile compounds. HCA was performed using the Metabo Analyst 2.0 software package (www.metaboanalyst.ca). All volatile data were log10-transformed and used in the HCA analysis. PCA was processed using the XLSTAT (Addinsoft, Paris, France) software package.

## 4. Conclusions

There are quantitative and qualitative differences of volatile compounds among Chinese mango cultivars, and a 123-fold difference (max. and min. ratio) in the quantity of volatiles evolved from different cultivars. Among the 37 germplasm resources, with the exception of the Boluoxiang cultivar, which has a fruit aroma which is primarily dependent on esters, the fruit aromas of other cultivars are mainly dependent on monoterpenes and sesquiterpenes, followed by non-terpenoid hydrocarbons and esters, while lower diversity and concentrations of aldehydes, alcohols, and ketones are present in the fruits. Notably, the fruits of certain cultivars such as Xiaofei, Guire10, Jinhuang, Guire7 and Guixiang, possess a greater diversity and higher concentrations of volatile aromatic compounds. All the cultivars could be divided into four groups using HCA and PCA. In conclusion, this study provides a detailed database of volatile composition of Chinese mango germplasm, which can be used for breeding a more diversified set of mango flavourswhich will eventually satisfy our diet and industrial production.

## Figures and Tables

**Table 1:** Volatiles detected in fruits of all 37 cultivars.

Monoterpene	Code	Non-Terpene Hydrocarbon	Code	Decanoic Acid Ethyl Ester	E11
α-pinene	M1	Styrene	H1	Butanoic acid ethyl ester	E12
β-Ocimene	M2	2,4-dimethyl-Heptane	H2	Butanoic acid butyl ester	E13
Limonene	M3	Decane	H3	3-Hexen-1-ol acetate	E14
Terpinene	M4	Nonadecane	H4	Oxalic acid, 6-ethyloct-3-yl isohexyl ester	E15
β-Myrcene	M5	Tetradecane	H5	Hexanoic acid ethyl ester	E16
α-Terpinene	M6	Hexadecane	H6	Octanoic acid methyl ester	E17
3-Carene	M7	Heptadecane	H7	Propanoic acid 2-methyl-3-methylbutyl ester	E18
(1S)-(+)-3-Carene	M8	Octacosane	H8	Butanoic acid propyl ester	E19
γ-Terpinene	M9	2,4,6-trimethyl-Octane	H9	Ethyl 2-hexenoate	E20
4-carene	M10	Heptacosane	H10	Butanoic acid octyl ester	E21
β-pinene	M11	2,6,10-trimethyl-Pentadecane	H11	Tetradecanoic acid ethyl ester	E22
Camphene	M12	Pentadecane	H12	Propyl octanoate	E23
2-Thujene	M13	1-Fluorononane	H13	3-Hydroxymandelic acid ethyl ester	E24
Sylvestrene	M14	Eicosane	H14	1,2-Benzenedicarboxylic acid mono(2-ethylhexyl) ester	E25
Ocimene	M15	Heneicosane	H15	Aldehyde	Code
α-Phellandrene	M16	1,5,9,9-tetramethyl-1,4,7-Cycloundecatriene	H16	Heptanal	A1
Neoalloocimene	M17	1,3,8-*p*-Menthatriene	H17	2,6-Nonadienal	A2
β-Pinene	M18	1,3,5,8-Undecatetraene	H18	13-Octadecenal	A3
D2-Carene	M19	10-Methylnonadecane	H19	Tetradecanal	A4
β-Terpinene	M20	1,5,5-Trimethyl-6-methylene-cyclohexene	H20	3,6-Nonadienal	A5
α-Pyronene	M21	1,3,5,7-Cyclooctatetraene	H21	2-Nonenal	A6
Artemisia triene	M22	5-Octadecene	H22	Nonanal	A7
1,3,5,5-tetramethyl-1,3-Cyclohexadiene	M23	2,2-dimethyl-3-methylene-Bicyclo[2.2.1]heptan	H23	Furfural	A8
Sesquiterpene	Code	2,6,10,15-tetramethyl-Heptadecane	H24	5-methyl-2-Furancarboxaldehyde	A9
Caryophyllene	S1	2,3,5-trimethyl-Decane	H25	5-Acetoxymethyl-2-furaldehyde	A10
α-Caryophyllene	S2	3-methyl-Dodecane	H26	Isopentyl hexanoate	A11
Germacrene D	S3	Undecane	H27	Alcohol	
α-Selinene	S4	1-methyl-2-(1-methylethyl)-Benzene	H28	2-propyl-1-Heptanol	B1
g-Selinene	S5	Tetratriacontane	H29	2-butyl-1-Octanol	B2
α-Gurjunene	S6	Ester	Code	1-Nonanol	B3
α-bulnsene	S7	Oxalic acid, isobutyl nonyl ester	E1	3,6-dimethoxy-9-(2-phenylethynyl)-Fluoren-9-ol	B4
Alloaromadendrene	S8	2-Propenoic acid, 2-ethylhexyl ester	E2	4-Ethyl-1-hexyn-3-ol	B5
α-Cubebene	S9	Oxalic acid, isohexyl pentyl ester	E3	Ketone	Code
Copaene	S10	Oxalic acid, isobutyl pentyl ester	E4	4-(2,6,6-trimethyl-1-cyclohexen-1-yl)-3-Buten-2-one	K1
β-Elemene	S11	2-Propenoic acid, 6-methylheptyl ester	E5	4-methoxy-2,5-dimethyl-3(2H)-Furanone	K2
Cubebene	S12	Oxalic acid, allyl nonyl ester	E6	1-(1,4-dimethyl-3-cyclohexen-1-yl)-Ethanone	K3
Calarene	S13	Dodecanoic acid, ethyl ester	E7	12-methyl-Oxacyclododec-9-en-2-one	K4
Epi-bicyclosesquiphellandrene	S14	Butanoic acid, 3-hexenyl ester	E8	2,3-dihydro-3,5-dihydroxy-6-methyl-4H-Pyran-4-one	K5
Isoledene	S15	Butanoic acid, hexyl ester	E9		
Aromadendrene	S16	Octanoic acid, ethyl ester	E10		

**Table 2:** Concentrations (μg/kg fresh weight (FW) equivalent of nonyl acetate) of volatile monoterpenes in 37 mango cultivars.

	1	2	3	4	5	6	7	8	9	10	11	12	13	14	15	16	17	18	19
M1	3.48	7.95	56.77	84.52	263.53	581.23	39.28	3.14	5.02	551.59	7.34	5.06	416.76	9.5	25.54	1023.02	15.65	112.35	962.28
M2	24.23	-	14.7	3.92	-	6.86	50.78	-	1.76	23.08	3.43	-	2.91	-	-	439.03	1.57	172.44	345.75
M3	6.73	-	-	-	78.24	-	18.1	-	-	10,565.52	-	19.02	202	-	75.88	58.55	-	-	49.54
M4	19.73	756.93	8457.23	2339.88	107.19	506.2	23.55	17.58	23.57	33.15	923.16	20.73	260.25	1464.44	127	21.33	1664.68	12,725.64	21.04
M5	-	13.81	93.85	-	41.39	244.45	-	-	-	120.03	-	12.31	108.05	-	-	3502.42	23.17	184.04	5557.77
M6	-	81.25	297.25	154.56	-	42.33	-	24.32	59.75	19.98	34.24	-	-	71.1	-	27.06	65.61	577.17	-
M7	-	102.24	175.02	-	1843.72	7704.67	-	572.22	763.84	-	80.16	504.67	4086.38	147.86	2326.81	-	-	-	-
M8	-	54.19	717.56	1297.64	-	-	-	444.71	-	-	-	566.63	-	0	1641.38	-	594.49	-	-
M9	-	38.92	217.64	72.99	10.54	34.47	-	-	-	39.99	-	-	20.99	47.17	-	26.04	8.71	1192.2	25.53
M10	-	-	3816.43	1365.48	-	-	-	-	-	-	-	-	-	-	-	-	71.38	70.3	-
M11	-	-	-	-	15.4	71.05	-	-	-	-	-	-	41.26	-	-	123.45	-	2.32	-
M12	-	-	-	-	-	13.53	-	-	-	-	-	-	5.14	-	-	16.87	-	-	13.89
M13	-	-	-	65.29	-	-	5453.71	-	-	-	-	-	-	-	42.88	-	-	141.94	5739.61
M14	-	-	-	-	-	335.18	-	16.89	0	-	29.3	-	-	-	-	-	-	-	-
M15	-	-	-	-	-	12.01	-	-	30.63	-	-	-	-	14.63	-	-	-	-	14.05
M16	-	-	-	-	-	0	-	-	-	40.36	-	-	-	-	-	-	-	-	-
M17	-	-	-	-	-	0	-	-	-	8.07	-	-	-	-	-	400.84	-	-	280.91
M18	-	-	-	-	37.47	176.39	-	-	-	-	-	-	104.18	39.65	30.91	152.19	29.61	-	104.79
M19	-	-	-	-	8.01	-	-	-	-	-	-	-	19.19	1610.71	-	12.18	-	-	-
M20	-	-	-	-	-	-	-	-	16.47	-	-	-	-	45.04	-	-	-	601.89	-
M21	-	-	-	-	-	-	-	-	-	-	-	-	-	-	-	-	-	7.99	-
M22	-	-	-	-	-	-	-	-	-	-	-	-	-	-	-	-	-	-	-
M23	-	-	-	-	-	-	-	-	-	-	-	-	-	-	-	-	-	-	-
Subtotal	54.17	1055.29	13,846.46	5447.44	-	9728.38	5585.43	1078.86	901.04	11,571.28	1077.65	1128.42	-	3450.10	4270.41	5802.99	2474.87	15,788.29	13,115.16
	**20**	**21**	**22**	**23**	**24**	**25**	**26**	**27**	**28**	**29**	**30**	**31**	**32**	**33**	**34**	**35**	**36**	**37**	
M1	511.88	4.76	122.39	104.44	5.68	11.23	17.86	55.29	1661.56	25.5	2.43	12.71	17.43	66.83	76.25	34.94	167.23	15.31	
M2	1065.36	-	98.58	160.64	-	-	-	-	12.68	-	-	-	75.43	44.57	306.66	21.03	1125.21	-	
M3	47.54	-	6.19	7.14	-	-	-	-	-	-	-	-	-	-	-	-	-	-	
M4	7.99	29.59	8.36	7.65	687.93	92.54	1000.18	6675.97	4.64	3192.13	7343.6	1554.95	1.22	7411.5	8703.66	933.59	6.28	496.25	
M5	3257.4	10.74	234.33	-	-	28.79	36.64	80.65	-	0	89.25	19.33	-	96.28	100.72	-	18.29	-	
M6	-	43.59	-	-	27.38	67.4	105.69	312.37	-	203.43	356.02	73.99	-	388.17	296.43	23.64	-	17.92	
M7	-	663.45	-	-	63.97	1436.92	115.54	10.84	-	4.92	51.74	167.66	-	-	692.02	72.22	44.98	23.06	
M8	-	-	-	-	-	932.55	267.87	-	-	1220.36	36.5	-	-	693.51	-	-	-	34.69	
M9	28.27	-	-	-	-	-	91.91	581.44	-	18.78	651.32	141.31	-	767.38	477.88	-	-	-	
M10	-	-	-	-	-	-	2940.17	-	-	64.63	-	-	-	13,945.6	-	33.57	-	-	
M11	68.61	-	-	8.79	6.93	-	-	-	9.92	-	-	-	-	-	-	-	-	-	
M12	10.69	-	-	-	-	-	-	-	0	-	-	-	-	-	-	-	-	-	
M13	3860.6	0	426.31	378.89	-	-	-	-	1310.38	55.04	113.27	-	-	-	78.66	-	-	7.61	
M14	-	-	-	-	-	-	-	-	-	-	-	-	-	-	-	-	-	-	
M15	-	-	-	-	-	-	-	-	-	-	-	-	-	-	-	4.12	6.41	-	
M16	-	-	-	-	-	-	-	-	-	-	-	-	-	-	-	-	-	-	
M17	940.89	1.46	149.18	155.33	-	-	-	-	-	-	-	-	-	-	24.35	-	20.68	-	
M18	70.7	9.57	16.18	12.71	-	-	-	-	1557.46	-	-	19.63	-	-	98.67	6.42	6.39	-	
M19	-	-	-	-	-	6.29	-	-	-	-	-	2195.17	-	-	-	-	-	-	
M20	-	-	-	-	24.4	32.89	121	221.61	-	180.05	257.21	44.26	0	298.06	300.1	-	-	-	
M21	-	-	-	-	-	-	-	3.29	-	-	-	-	-	-	-	-	4.98	-	
M22	5.73	-	-	-	-	-	-	-	-	-	-	-	-	10.74	-	-	-	-	
M23	-	-	-	-	-	-	-	2.96	-	0.92	4.01	-	-	3.97	-	-	-	-	
Subtotal	9875.68	763.16	1061.52	835.59	816.29	2608.61	4696.86	7944.42	4556.64	4965.76	8905.35	4229.01	94.08	23,726.61	11,155.40	1129.53	1400.45	594.84	

Numbers 1–37 represent the cultivars responding to the accession number in Table 2; The letter plus the number represent the compound corresponding to the code in Table 1;—Indicated that the compound was not detected; The same as below.

**Table 3:** Concentrations (μg/kg fresh weight (FW) equivalent of nonyl acetate) of volatile sesquiterpenes in 37 mango cultivars.

	1	2	3	4	5	6	7	8	9	10	11	12	13	14	15	16	17	18	19
S1	445.68	20.93	84.46	15.04	-	293.95	61.65	22.28	33.38	125.14	41.45	11.66	167.24	20.44	106.89	52.46	11.75	21.49	319.07
S2	255.8	9.55	-	4.31	-	-	40.07	9.99	22.93	49.9	-	-	88.71	6.09	54.22	24.49	4.04	3.89	160.6
S3	4.59	-	-	-	-	177.13	-	-	-	36.44	5.87	-	76.74	-	63.46	17.7	5.86	-	-
S4	229.68	104.43	-	-	1.48	94.89	4.68	2.57	-	49.75	161.95	-	22.85	-	8.6	15.86	5.38	250.69	-
S5	26.22	-	-	-	-	-	-	-	-	29.05	-	-	-	-	-	-	-	9.15	-
S6	-	7.64	16.75	-	-	21.51	-	2.95	-	-	-	-	-	-	-	-	-	20.31	54.32
S7	-	9.19	-	-	12.2	102.39	-	-	-	74.4	12.24	-	38.16	-	8.16	24.93	10.63	-	-
S8	-	-	-	-	4.43	13.85	-	-	-	-	-	-	19.47	-	4	11.09	2.69	7.83	4.02
S9	-	-	-	-	9.26	12.72	-	-	-	-	-	-	0	-	-	-	2.09	73.4	-
S10	-	-	-	-	6.92	31.84	142.7	-	-	17.14	-	-	11.14	-	-	10.14	-	57.36	-
S11	-	-	-	-	-	6.65	-	-	-	-	-	-	0	-	-	-	-	-	-
S12	-	-	-	-	-	29.95	-	-	-	16.1	-	-	14.46	-	10.95	3.97	-	-	-
S13	-	-	-	-	-	16.83	-	-	-	6.94	-	-	11.37	-	-	-	-	-	-
S14	-	-	-	-	-	11.58	-	-	-	-	-	-	7.72	-	-	-	-	-	-
S15	-	-	-	-	-	39.38	-	-	-	11.19	-	-	0	-	-	-	-	-	-
S16	-	-	-	-	-	11.85	-	-	-	-	-	-	6.31	-	-	-	-	-	-
Subtotal	961.96	151.74	101.22	19.35	34.28	864.55	249.11	37.79	56.31	416.05	221.51	11.66	464.17	26.52	256.28	160.64	42.44	444.12	538.01
	**20**	**21**	**22**	**23**	**24**	**25**	**26**	**27**	**28**	**29**	**30**	**31**	**32**	**33**	**34**	**35**	**36**	**37**	
S1	45.84	18.27	14.96	26.18	0	18.21	13.67	22.76	61.23	33.77	9.37	-	-	128.41	80.81	-	25.42	6.35	
S2	28.86	10.46	0	17.85	0	11.1	0	13.46	32.15	21.33	4.85	-	-	53.67	44.76	-	-	2.29	
S3	-	-	-	-	-	-	-	-	-	-	4.33	-	-	108.29	-	-	-	-	
S4	-	-	-	-	35.15	-	45.89	-	14.35	-	-	-	-	35.49	-	-	-	34.65	
S5	-	-	-	-	-	-	-	-	-	-	-	-	-	-	-	-	-	-	
S6	4.18	-	-	2.35	3.74	-	4.04	-	-	-	-	-	-	-	5.08	-	-	2.84	
S7	-	-	-	-	-	-	-	-	-	-	-	-	-	-	-	-	21.38	-	
S8	-	-	-	-	-	-	-	-	-	-	-	-	-	-	-	-	10.96	-	
S9	-	-	-	-	-	-	-	-	-	-	23.49	-	-	5.84	-	-	-	-	
S10	-	-	-	-	-	-	-	-	-	-	10.31	-	-	6.36	-	-	-	-	
S11	-	-	-	-	-	-	-	-	-	-	-	-	-	15.69	-	-	-	-	
S12	-	-	-	-	-	-	-	-	-	-	5.29	-	-	23.32	-	-	-	-	
S13	-	-	-	-	-	-	-	-	-	-	-	-	-	10.36	-	-	2.89	-	
S14	-	-	-	-	-	-	-	-	-	-	-	-	-	16.07	-	-	-	-	
S15	-	-	-	-	-	-	-	-	-	-	-	-	-	-	-	-	-	-	
S16	-	-	-	-	-	-	-	-	-	-	-	-	-	-	-	-	-	-	
Subtotal	78.88	28.73	14.96	46.38	42.25	29.31	63.60	36.22	107.73	55.10	57.64	-	-	403.50	130.65	-	60.65	46.13	

**Table 4:** Concentrations (μg/kg fresh weight (FW) equivalent of nonyl acetate) of volatile non-terpene hydrocarbons in 37 mango cultivars.

	1	2	3	4	5	6	7	8	9	10	11	12	13	14	15	16	17	18	19
H1	5.66	-	-	8.97	9.29	10.81	-	4.62	3.67	4.4	10.27	7.15	9.91	-	7.42	14.05	5.48	7.97	-
H2	2.44	-	4.36	-	2.16	-	-	-	7.8	1.21	2.31	-	-	-	-	-	-	-	-
H3	6.05	-	-	-	-	-	-	3.36	-	-	-	-	-	-	-	4.06	-	-	-
H4	33.45	14.67	18.09	5.17	4.72	-	-	-	25.33	10.41	-	32.22	6.23	8.62	18.81	4.13	8.31	13.55	4.76
H5	10.5	12.52	8.61	-	-	-	-	7.88	11.63	6.21	-	4.94	-	22.41	13.3	-	10.96	12.91	-
H6	-	-	-	-	-	-	-	-	-	66.12	-	-	10	6.87	-	8.72	-	-	-
H7	8.3	6.01	-	-	-	-	-	-	9.19	12.99	-	-	-	22.71	-	-	-	-	-
H8	92.44	16.2	41.99	-	-	41.16	-	17.92	64.66	28.72	-	4.05	16.66	20.99	54.1	15.96	32.87	-	-
H9	-	-	-	5.79	-	-	-	0	0	10.86	-	28.64	-	-	-	-	5.01	-	-
H10	7.64	6.95	7.31	15.99	-	-	-	10.71	26.16	14.27	-	4.13	16.33	33.95	20.12	-	36.48	-	-
H11	10.94	-	-	-	-	-	-	0	13.28	14.03	-	-	-	-	10.28	-	-	-	-
H12	2.03	-	4.18	4.01	7.23	0	-	7.11	14.1	0	-	12.05	-	-	6.86	-	6.81	-	-
H13	-	3.49	7.52	-	15.55	2.55	-	0	2.62	0	-	-	-	-	-	-	-	-	-
H14	-	-	8.7	-	5.48	-	-	3.31	5.55	11.76	-	-	-	-	-	-	-	-	-
H15	-	-	22.08	-	-	-	-	0	53.86	10.59	-	30.07	-	22.65	-	-	-	-	-
H16	-	-	53.82	-	-	132.53	-	13.47	20.18	143.79	5.15	6.18	-	14.37	-	30.17	-	-	-
H17	-	-	28.72	5.72	-	-	-	-	-	-	-	-	-	-	-	17.36	-	9.88	-
H18	-	-	6.16	-	-	-	2.88	-	-	-	-	-	-	-	-	3.18	-	-	-
H19	-	33.26	-	11.61	-	-	-	-	23.98	-	-	-	-	-	12.89	-	20.86	-	17.13
H20	-	-	-	-	-	17.61	-	-	-	-	-	-	12.37	-	13.69	-	-	-	-
H21	-	3.26	3	-	-	-	10.72	3.35	3.95	-	-	-	11.63	-	3.6	-	5.43	7.68	12.34
H22	-	-	-	-	-	-	-	-	7.97	-	-	-	-	-	-	-	-	-	-
H23	-	-	-	-	-	10.83	-	-	-	-	31.89	-	7.52	-	-	22.94	-	-	-
H24	-	-	-	-	-	-	-	-	-	-	-	-	-	-	10.93	-	-	-	-
H25	-	-	-	4.75	-	-	-	-	16.2	9.89	-	-	-	-	-	-	6.38	-	10.68
H26	-	-	-	-	-	-	-	-	31.5	21.85	-	-	-	-	6.22	-	-	-	-
H27	-	-	5.03	4.75	14.52	-	-	7.04	22.7	14.01	-	9.06	2.96	-	15.6	-	10.96	-	12.11
H28	-	-	104.75	-	-	-	-	-	-	6.82	5.76	-	-	-	-	-	-	-	-
H29	-	-	-	-	-	-	-	-	13.28	56.11	-	-	5.82	-	-	-	-	-	-
Subtotal	179.45	96.37	324.34	66.75	58.97	215.49	13.60	78.77	377.61	444.04	55.38	138.50	99.43	152.57	193.81	120.57	149.55	51.98	57.03
	**20**	**21**	**22**	**23**	**24**	**25**	**26**	**27**	**28**	**29**	**30**	**31**	**32**	**33**	**34**	**35**	**36**	**37**	
H1	6.72	2.56	16.52	4.66	6.15	7.57	8.88	8.42	6.8	6.14	9.01	7.88	5.18	7.4	3.52	4.72	17.62	6.09	
H2	-	1.64	-	3.14	5.04	-	2.09	8.78	2.53	-	5.54	-	3.56	-	3.12	-	-	3.25	
H3	4.54	4.24	-	-	4.84	-	3.44	6.81	-	-	-	3.43	6.9	5.66	4.51	11.74	4.83	-	
H4	15.81	10.53	-	26.32	-	11.66	5.26	16.92	-	8.99	9.97	10.93	-	39.81	13.57	8.67	15.81	4.56	
H5	9.28	8.71	11.36	15.82	8.74	8.99	-	13.76	5.74	9.09	9.26	11.34	9.61	12.83	9.2	10.6	-	-	
H6	7.2	26.47	-	15	-	12.44	-	14.72	-	6.34	18.04	10.48	8.69	27.13	7.32	11.73	24.68	-	
H7	10.22	24.71	-	7.4	-	-	-	12.73	-	14.03	14.39	-	-	-	12.28	14.78	-	-	
H8	20.52	44.81	-	73.5	-	22.81	-	44.2	-	36.71	27.62	11.23	19.16	18.11	-	38.27	-	-	
H9	-	-	2.46	-	-	11.96	-	-	-	-	6.36	-	6.24	0	-	-	-	-	
H10	19.48	10.44	-	11.21	-	29.87	-	16.74	-	16.84	-	-	-	47.77	-	-	19.97	-	
H11	-	-	-	4.22	-	-	-	-	-	8.55	8.59	8.14	-	4.92	-	3.98	-	-	
H12	21.16	-	-	5.73	-	7.12	-	6.6	-	8.19	4.46	-	9.03	30.59	-	-	-	2.03	
H13	6.07	-	-	-	-	4.9	7.83	-	-	-	-	-	4.84	-	3.7	-	-	11.67	
H14	-	2.87	5.47	18.12	-	-	-	7.28	-	2.82	5.7	18.71	-	-	-	-	-	-	
H15	15.23	9.31	17.18	13.43	-	14.99	-	26.15	-	14.57	24.3	54.47	13.73	11.58	15.21	-	-	-	
H16	-	-	20.13	10.24	-	8.83	5.85	-	-	21.98	6.32	-	-	-	44.1	-	12.77	-	
H17	-	-	-	-	4.14	-	-	22.39	-	-	8.18	-	-	45.87	31.51	-	-	-	
H18	-	-	4.55	-	-	-	-	-	-	-	-	-	-	-	4.26	-	-	-	
H19	17.1	17.35	-	-	-	-	-	13.79	19.2	5.86	10.42	36.55	-	11.52	77.77	-	-	-	
H20	-	-	-	-	-	-	-	-	-	-	-	-	-	-	-	-	-	-	
H21	-	-	-	-	6.29	-	-	2.59	5.56	-	-	8.84	-	14.52	7.02	-	15.94	-	
H22	-	2.91	-	-	-	-	-	-	-	-	-	-	-	-	-	-	-	-	
H23	10.05	-	-	-	-	-	-	-	-	-	-	-	-	322.72	-	-	-	-	
H24	-	3.14	-	6.18	-	5.18	-	5.58	-	-	-	-	-	-	-	-	-	-	
H25	12.78	-	-	8.3	-	5.95	-	-	-	15.72	15.98	-	2.39	-	20.47	-	-	-	
H26	6.45	9.96	-	22.15	-	-	-	-	-	-	-	-	-	13.33	-	-	-	-	
H27	5.8	14.28	-	4.41	8.07	10.28	-	4.62	-	15.33	7.79	-	9.51	-	-	11.5	30.16	2.81	
H28	-	-	-	-	-	-	9.32	-	-	-	-	24.77	-	118.18	117.53	6.5	3.48	3.43	
H29	-	12.52	-	13.1	-	-	-	-	6.28	-	-	-	7.24	12.19	-	21.84	21.66	-	
Subtotal	188.42	206.45	77.67	262.93	43.27	162.55	42.67	232.08	46.11	191.16	191.93	206.77	106.08	744.13	375.09	144.33	166.92	33.84	

**Table 5:** Concentrations (μg/kg fresh weight (FW) equivalent of nonyl acetate) of volatile aldehydes in 37 mango cultivars.

	1	2	3	4	5	6	7	8	9	10	11	12	13	14	15	16	17	18	19	20	21	22
A1	-	-	-	-	-	3.92	-	-	-	-	-	4.41	3.42	-	-	7.16	-	-	-	-	-	-
A2	-	-	-	-	2.42	14.29	-	-	18.57	-	-	-	-	-	-	-	14.03	-	19.82	11.75	-	-
A3	-	-	-	-	-	-	77.77	-	-	-	-	-	-	-	-	-	-	-	-	-	-	-
A4	-	-	-	-	-	-	63.52	-	-	-	-	-	-	-	-	-	-	-	-	-	-	-
A5	-	-	-	-	-	-	-	-	8.06	-	-	-	-	-	-	-	-	-	-	-	3.76	-
A6	-	-	-	-	-	-	-	-	-	-	-	12.5	-	-	-	-	-	-	-	-	-	-
A7	-	-	-	-	-	-	-	-	6.84	-	-	-	-	-	7.22	-	-	-	-	-	7.38	-
A8	-	-	-	-	-	-	-	-	-	-	-	-	-	-	-	-	-	-	-	-	-	168.9
A9	-	-	-	-	-	-	-	-	-	-	-	-	-	-	-	-	-	-	-	-	-	566.11
A10	-	-	-	-	-	-	-	-	-	-	-	-	-	-	-	-	-	-	-	-	-	12.39
A11	-	-	-	-	-	-	-	-	-	-	-	-	-	-	-	-	-	-	-	-	-	-
Subtotal	-	-	-	-	2.42	18.21	141.29	-	33.48	-	-	16.91	3.42	-	7.22	7.16	14.03		19.82	11.75	11.14	747.40
	**23**	**24**	**25**	**26**	**27**	**28**	**29**	**30**	**31**	**32**	**33**	**34**	**35**	**36**	**37**							
A1	5.3	-	-	-	-	10.66	-	-	-	-	-	-	-	-	-							
A2	4.91	-	14.02	-	-	-	-	108.12	-	-	-	-	-	-	-							
A3	-	-	-	-	-	-	-	-	-	-	-	-	-	-	-							
A4	-	-	-	-	-	-	-	-	-	-	-	-	-	-	-							
A5	-	-	5.26	-	-	-	-	35.25	-	-	-	-	-	-	-							
A6	-	-	-	-	-	-	-	-	-	-	-	-	-	-	-							
A7	11.61	-	-	-	-	8.51	18.46	18.28	-	-	-	-	-	-	-							
A8	-	-	-	-	-	-	-	-	-	-	-	-	-	-	-							
A9	-	-	-	-	-	-	-	-	-	-	-	-	-	-	-							
A10	-	-	-	-	-	-	-	-	-	-	-	-	-	-	-							
A11	-	-	-	-	-	-	-	-	-	-	44.61	-	-	-	-							
Subtotal	21.82	-	19.28	-	-	19.17	18.46	161.65		-	44.61											

**Table 6:** Concentrations (μg/kg fresh weight (FW) equivalent of nonyl acetate) of volatile esters in 37 mango cultivars.

	1	2	3	4	5	6	7	8	9	10	11	12	13	14	15	16	17	18	19
E1	2.59	1.59	13.17	-	2.68	-	-	3.39	-	-	-	4	2.79	-	-	3.65	-	7.53	6.48
E2	2.69	-	-	2.07	2.35	-	-	1.92	-	1.62	-	2.77	-	-	2.66	2.49	2.39	-	3.1
E3	-	4.88	-	-	-	-	-	7.18	2.01	0.72	3.19	-	-	-	-	-	4.71	-	-
E4	-	-	-	5.35	1.91	1.25	2.23	-	-	-	2.52	2.18	2.34	2.81	-	-	1.42	2.48	-
E5	-	1.43	-	-	-	2.37	2.71	-	-	-	0	0	2.33	3.64	-	-	2.38	2.23	-
E6	-	3.36	-	-	3.79	-	-	-	-	-	-	-	-	-	2.89	-	-	-	-
E7	-	-	22.02	-	-	-	-	-	-	-	-	-	-	-	56.69	-	-	-	-
E8	-	-	5.02	-	15.04	3.29	43.13	7.15	-	34.21	-	-	-	-	10.56	-	-	-	-
E9	-	-	57.9	-	-	-	-	6.72	-	-	-	-	-	-	140.4	-	-	-	-
E10	-	-	7.8	-	-	3.63	9.16	-	-	-	-	-	-	-	1699.42	-	-	-	10.35
E11	-	-	15.31	-	-	-	-	-	-	-	-	-	-	-	88.04	-	-	-	-
E12	-	-	81.85	-	-	21.97	-	-	-	-	-	-	-	-	2916.45	-	-	-	-
E13	-	-	157.96	-	-	-	-	-	-	-	-	-	-	-	-	-	-	-	-
E14	-	-	-	-	-	-	97	-	-	-	-	-	-	-	-	-	-	-	-
E15	-	3.04	-	-	-	-	-	4.43	-	-	-	9.37	-	-	2.96	-	2.98	-	-
E16	-	-	-	-	-	-	-	-	-	-	-	-	-	1.11	273.04	-	-	-	-
E17	-	-	-	-	-	-	-	-	-	-	-	-	-	-	21.28	-	-	-	-
E18	-	-	-	-	-	-	-	-	-	-	-	-	-	-	29.01	-	-	-	-
E19	-	-	-	-	-	-	-	-	-	-	-	-	-	-	29.63	-	-	-	-
E20	-	-	-	-	-	-	-	-	-	-	-	-	-	-	18.57	-	-	-	-
E21	-	-	-	-	-	-	-	-	-	-	-	-	-	-	34.42	-	-	-	-
E22	-	-	-	-	-	-	-	-	-	-	-	-	-	-	20.2	-	-	-	-
E23	-	-	-	-	-	-	-	-	-	-	-	-	-	-	10.49	-	-	-	-
E24	-	-	-	-	-	-	-	-	-	-	-	-	-	-	4.4	-	10.16	-	-
E25	-	-	-	-	-	-	-	-	-	-	-	-	-	-	-	-	-	-	-
Subtotal	5.28	14.31	361.05	7.42	25.78	32.51	154.23	30.78	2.01	36.55	5.71	21.72	7.45	7.56	5361.09	6.14	24.04	12.24	19.93
	**20**	**21**	**22**	**23**	**24**	**25**	**26**	**27**	**28**	**29**	**30**	**31**	**32**	**33**	**34**	**35**	**36**	**37**	
E1	-	-	-	-	-	-	-	-	-	-	-	-	2.41	-	-	-	-	-	
E2	-	1.38	3.34	2.26	1.7	1.86	-	2.07	-	2.96	2.41	-	1.46	-	-	-	3.48	2.18	
E3	5.42	-	-	-	-	-	-	-	-	6.65	2.79	6.09	-	-	-	3.04	-	-	
E4	-	2.22	-	-	-	1.11	-	-	-	1	2.34	-	1.38	-	-	-	-	-	
E5	1.96	-	-	-	-	-	2.44	-	1.84	2.41	-	-	1.61	3.01	2.81	-	3.73	-	
E6	-	3.88	-	-	-	4.03	-	2.95	-	-	4.15	4.02	3.23	-	-	-	-	-	
E7	-	-	-	-	28.84	-	-	26.13	-	-	-	-	-	68.19	37.02	-	-	-	
E8	-	-	-	4.57	-	-	-	0	19.93	-	-	15.36	-	-	8.3	14.59	9.99	-	
E9	-	-	-	-	-	-	-	5.97	43.61	-	-	7.63	-	116.78	167.06	-	-	-	
E10	2.16	-	-	-	-	-	-	0	26.89	-	-	3.71	-	11.84	27.66	-	-	-	
E11	-	-	-	-	12.96	-	-	7.62	5.12	-	-	11.79	-	42.69	27	-	-	-	
E12	-	-	-	-	22.14	-	-	19.17	303.46	-	-	21.9	-	117.21	41.73	-	-	-	
E13	-	-	-	-	-	-	-	-	-	-	-	-	-	277.79	214	-	10.85	-	
E14	-	-	-	-	-	-	-	-	-	-	-	-	-	-	-	-	-	-	
E15	-	-	-	-	3.56	-	-	-	-	-	-	-	-	-	-	-	-	-	
E16	-	-	-	-	-	-	-	-	-	-	-	-	-	-	-	-	-	-	
E17	-	-	-	-	-	-	-	-	-	-	-	-	-	-	-	-	-	-	
E18	-	-	-	-	-	11.48	-	-	36.75	-	-	-	-	310.07	-	12.18	-	-	
E19	-	-	-	-	-	-	-	-	-	-	-	-	-	-	-	-	-	-	
E20	-	-	-	-	-	-	-	-	-	-	-	-	-	-	-	-	-	-	
E21	-	-	-	-	-	-	-	-	-	-	-	-	-	113.3	-	-	-	-	
E22	-	-	-	-	11.42	-	-	-	-	-	-	-	-	42.18	-	-	-	-	
E23	-	-	-	-	-	-	-	-	-	-	-	-	-	-	-	-	-	-	
E24	-	1.02	-	6.19	-	-	-	-	4.46	7.36	-	-	0.76	-	-	-	7.92	-	
E25	-	-	-	-	-	-	-	39.05	-	-	23.32	-	-	-	-	-	-	-	
Subtotal	9.54	8.50	3.34	13.02	80.62	18.48	2.44	102.96	442.06	20.38	35.01	70.50	10.85	1103.06	525.58	29.81	35.97	2.18	

**Table 7:** Concentrations (μg/kg fresh weight (FW) equivalent of nonyl acetate) of volatiles alcohols and ketones in 37 mango cultivars.

	1	2	3	4	5	6	7	8	9	10	11	12	13	14	15	16	17	18	19	20	21	22
B1	14.36	-	-	-	-	-	-	-	-	-	-	-	-	-	-	0	17.25	-	-	21.75	9.3	-
B2	-	16.24	-	-	-	-	-	-	-	-	-	-	-	-	-	-	-	7.27	-	-	-	-
B3	-	-	22.27	-	-	-	5.74	-	2.66	-	-	3.58	-	-	4.72	-	-	3.29	-	-	-	4.47
B4	-	-	-	-	-	-	-	-	-	-	-	-	-	-	-	-	-	-	-	-	-	-
B5	-	-	-	-	-	-	-	-	-	-	-	-	-	-	-	-	-	-	-	-	-	-
Subtotal	14.36	16.24	22.27	-	-	-	5.74	-	2.66	-	-	3.58	-	-	4.72	-	17.25	10.56	-	21.75	9.30	4.47
	**23**	**24**	**25**	**26**	**27**	**28**	**29**	**30**	**31**	**32**	**33**	**34**	**35**	**36**	**37**							**-**
B1	-	-	25.01	-	-	17.38	-	-	-	-	-	-	29.03	-	19.46							
B2	-	-	-	-	-	-	-	-	-	-	-	-	-	-	-							
B3	-	-	-	8.3	-	-	3.7	7.04	-	-	-	-	-	13.23	-							
B4	-	-	-	-	3.39	-	-	3.32	2.73	-	-	-	-	-	-							
B5	-	-	-	-	0	11.84	-	-	-	-	-	-	-	-	-							
Subtotal	-	-	25.01	8.30	3.39	29.22	3.70	10.36	2.73	-	-	-	29.03	13.23	19.46							
	**1**	**2**	**3**	**4**	**5**	**6**	**7**	**8**	**9**	**10**	**11**	**12**	**13**	**14**	**15**	**16**	**17**	**18**	**19**	**20**	**21**	**22**
K1	-	-	-	-	-	-	19.01	-	-	-	-	-	-	-	-	-	-	-	-	-	-	-
K2	-	-	-	-	-	-	82.42	-	-	-	-	-	-	-	-	-	-	-	-	-	-	-
K3	-	-	-	-	-	-	4.08	-	-	-	-	-	-	-	-	-	-	-	-	-	-	-
K4	-	-	-	-	-	-	0	-	-	-	-	-	-	-	19.92	-	-	-	-	-	-	-
K5	-	-	-	-	-	-	0	-	-	-	-	-	-	-	0	-	-	-	-	-	-	60.27
Subtotal	-	-	-	-	-	-	105.51	-	-	-	-	-	-	-	19.92	-	-	-	-	-	-	60.27
	**23**	**24**	**25**	**26**	**27**	**28**	**29**	**30**	**31**	**32**	**33**	**34**	**35**	**36**	**37**							
K1	-	-	-	-	-	-	-	-	-	-	-	-	-	-	-							
K2	-	-	-	-	-	-	-	-	-	-	-	-	-	-	-							
K3	-	-	-	-	-	-	-	-	-	-	-	-	-	-	-							
K4	-	-	-	-	-	-	-	-	-	-	-	-	-	-	-							
K5	-	-	-	-	-	-	-	-	-	-	-	-	-	-	-							
Subtotal	42.00	-	-	-	-	-	-	-	-	-	-	-	-	-	-							

**Table 8:** Linear correlation coefficients among different classes of volatile compounds.

	Monoterpene	Sesquiterpene	Non-Terpene Hydrocarbons	Ester	Aldehyde	Alcohol	Ketone
**Monoterpene**	1						
**Sesquiterpene**	0.374 *	1					
**Non-Terpene Hydrocarbons**	0.569 **	0.159	1				
**Ester**	0.122	0.085	0.185	1			
**Aldehyde**	−0.076	−0.105	−0.090	−0.045	1		
**Alcohol**	−0.102	−0.143	−0.149	−0.046	−0.052	1	
**Ketone**	−0.104	−0.025	−0.167	0.106	0.565 **	−0.107	1

* Significant correlation at *p* < 0.05. ** Significant correlation at *p* < 0.01.

**Table 9:** 37 cultivars used in this study. The number following the cultivars indicates the sampling order.

**Cultivars**	814	Baili	Jinhuang	Duacan	Shixuan8	Shixuan5	Xingre	Shixuan7
**No** **.**	1	2	3	4	5	6	7	8
**Cultivars**	Hongjinfeng	Guixiang	Zaoshu	TA	Guangxi3	Aimang	Boluoxiang	Dasannian
**No.**	9	10	11	12	13	14	15	16
**Cultivars**	Dongzhen Hong	Guire10	Guire7	Sankeli	Hong6	Hongwa Cheng	Hutou	Huangyu
**No.**	17	18	19	19	20	21	22	23
**Cultivars**	Hongxiangya	Linsheng	Liuxian	Pingguo	Renong1	Tainong1	Lvsong	Shengshi
**No.**	25	26	27	28	29	30	31	32
**Cultivars**	Xiaofei	Xiaoji	Baodaohuang	Yingwu	Yuexi			
**No.**	33	34	35	36	37			
